# Chronic back pain and its association with quality of life in a large French population survey

**DOI:** 10.1186/s12955-018-1018-4

**Published:** 2018-09-26

**Authors:** Mathilde M. Husky, Farina Ferdous Farin, Philippe Compagnone, Christophe Fermanian, Viviane Kovess-Masfety

**Affiliations:** 1 Institut Universitaire de France, Laboratoire de Psychologie EA4139, Université de Bordeaux, Bordeaux, France; 2 Ecole des Hautes Etudes en Santé Publique, Paris, France; 3 Faculté de Psychologie, Université de Bordeaux, Bordeaux, France; 4 Laboratoire de Psychopathologie et Processus de Santé EA 4057, Université Paris Descartes, Sorbonne Paris Cité, Paris, France; 5 Ecole des Hautes Etudes en Santé Publique, Laboratoire de Psychopathologie et Processus de Santé EA 4057, Université Paris Descartes, Sorbonne Paris Cité, Paris, France

**Keywords:** Chronic pain, Back pain, Epidemiology, Quality of life

## Abstract

**Background:**

Chronic back pain is associated with significant burden, yet few epidemiological studies have provided data on chronic back pain, its predictors and correlates in France.

**Methods:**

Data were drawn from a cross-sectional survey conducted in France (*n* = 17,249) using computer-assisted telephone interviews. Sample age ranges from 18 to 98 with a mean of 46.39 years (SD = 17.44), and was 56.7% female. Medical conditions were assessed using the CIDI, quality of life was assessed using both the physical and mental component scores of the SF-36.

**Results:**

Overall, 38.3% of adults reported chronic back pain. Female gender, older age, lower education, manual labor occupation, and population density were significantly associated with the distribution of chronic back pain. Chronic back pain was associated with lower scores on all SF-36 mean scores and on the Physical Composite Score and Mental Composite Score controlling for comorbid medical conditions including other types of chronic pain.

**Conclusion:**

The study highlights the burden of chronic back pain in the general population and underscores its correlation with quality of life. Such data contribute to raise awareness among clinicians and health policy makers on the necessity of prevention, early diagnosis, proper management and rehabilitation policies in order to minimize the burden associated with chronic pain.

## Highlights

- Nearly 40% of general population adults reported chronic back pain.
- Female gender, older age, lower education, and rural areas were associated with increased risk.
- Chronic back pain is associated with significantly reduced quality of life.

## Background

Chronic pain has been defined as pain persisting beyond normal healing time and lasting usually for more than three to six months [1]. It has been estimated that one in every five adults suffers from chronic or recurring pain and another one in every 10 adults is newly diagnosed with chronic pain each year around the world [2]. Among all types of chronic pain, back pain has often been suggested to be the most frequent type of pain experienced [3–5]. In Europe, population-based surveys have shown that chronic pain essentially reflected back and lower back pain (24% and 18%, respectively) [6]. Chronic back pain has been found to be the leading cause of disability when analyzed globally and has remained one of the top two contributors of global disability causes for over two decades [7].

Musculoskeletal conditions such as back pain have a major impact on the health care system due to the combined high prevalence and associated disability. The total cost of back pain around the world is estimated to represent billions of dollars annually mainly due to indirect costs representing a large portion (75% to 93%) of the total costs of back pain [8–10]. While indirect cost reflects productivity loss and absenteeism, back pain also significantly limits daily activity by imposing functional limitations [11, 12]. Health Related Quality of Life (HRQL) is impacted by chronic back pain in different life domains such as physical and mental wellbeing, social relationships and functional ability [13]. Assessing HRQL can provide an estimate of how the disease influences people’s lives and how they manage to live with chronic back pain [14], and has become an important outcome in health care and as a measure of treatment efficacy [15]. Evaluating the quality of life of people with back pain is necessary in order to establish objectives and plan treatment, to monitor the evolution of pain and to assess the outcome of care both at the individual patient level as well as at the population level [16].

Few epidemiological studies have provided data on chronic back pain in France. The first study offered data collected in 2002–2003 in a national population survey on the general population (*n* = 14,248) between the ages of 30 and 64. The study estimated that more than 50% in this age group experienced lower back pain (LBP) at least one day in the previous 12 months. The prevalence of LBP was 17% when considering experiencing LBP for more than 30 days in the previous 12 months [17]. However the study focused on a limited age group and did not examine quality of life. A second general population survey was conducted in 15 European countries and Israel, and estimated at 15% the prevalence of chronic pain in France [6]. Among those who experienced chronic pain (*n* = 300), back (24%) and lower back pain (18%) were the most frequent types of pain. Furthermore, among chronic pain sufferers 59% were women, 15% had lost their employment due to their pain, and 67% reported inadequate pain management with medication. Country-specific information on correlates of back pain was, however, not presented. A third study stemmed from a large international collaborative study by the World Mental Health Survey (WMH) initiative and examined the comorbidity of chronic back pain and mental disorders, as well as service utilization and well-being [3]. The prevalence of back pain was 21,3%. However the French portion of the WMH sample was relatively small (*n* = 1436) and the associated factors were not presented. Taken together, the existing literature does not provide detailed information on the prevalence of chronic back pain and its association with socio-economic factors and quality of life in the French general population. Additional epidemiological data are needed to provide a better understanding of the prevalence and correlates of chronic pain in the country.

The present study uses a large population-based survey (1) to estimate the prevalence of chronic back pain in the general population; (2) to examine the association between chronic back pain and sociodemographic and clinical risk factors, and (3) to examine quality of life associated with chronic back pain.

## Methods

### Data and population

The present data were drawn from a large cross-sectional survey conducted in four regions of France: Normandy, Ile-de-France, Lorraine and Rhône-Alpes representing one third of the French population and largely diverse regions. Trained interviewers, using a computer-assisted telephone interviewing system, collected data between April and June 2005. In each region, participants were selected using a two-stage procedure. First, 59,836 households with landline numbers were randomly contacted. Second, one person was randomly selected within each household according to a method proposed by Kish [18]. Landline telephone numbers listed in the directory for each region were randomly chosen. The last digit of each number was then replaced with a randomly chosen number so as to include both listed and unlisted numbers. In addition to this sample, a mobile phone-only sample was collected in order to reach persons who were not equipped with a landline. Once the data were pooled, the final sample included 22,138 participants with an overall response rate of 62.7%. Exclusion criteria included being a non-French speaker, being a minor, being unable to answer the phone or complete the interview (i.e. the person suffered from deafness, did not answer the questions or answered inconsistently, was intoxicated, or suffered from a physical illness that prevented him or her from talking for a long period of time). Participants were given a complete description of the study and provided informed consent. The French ethics committee of the National Data Protection Authority (CNIL) approved recruitment and consent procedures. Interviews lasted an average of 37 minutes. In order to minimize survey duration, certain questions including questions regarding physical health problems were only asked to respondents who screened positive to a psychiatric screening question or screened positive for psychological distress based on the SF36 and to a random sample of one third of those who did not screen positive on any of the psychiatric diagnostic screening questions, and/or at any of the SF36 mental health scales. Of the 22,138 respondents, 7,270 screened negative on the initial mental health questions; accordingly one third (*n* = 2,381) of these respondents were administered the full interview while the remaining 4,889 were not. As a result, the present study is based on a total sample of 17,249 respondents who completed all sections of the survey. The data were weighted to account for the sampling strategy.

### Survey variables

#### Demographic and socioeconomic characteristics

Demographic and socioeconomic characteristics included: gender, age, educational attainment (no degree, middle school, high school, some college, college degree), employment status (employed, seeking employment, retired, student, housewife and other inactive) and type of occupation based on the national census classification [19] (farmer, independent, executive, intermediary profession, employee, blue collar worker, inactive), relationship status (single or in a relationship), size of city of residence (rural, < 20,000 inhabitants, 20,000 to 100,000, and > 200,000 inhabitants).

### Chronic pain

The CIDI chronic conditions module [20, 21] was used to assess past year physical illnesses or health events. Participants were asked whether or not they currently suffered or had suffered from any of 14 health problems in the previous year. Health problems were then regrouped in broader categories as follows: chronic pain (arthritis or rheumatism, chronic back or neck problems, frequent or severe headaches, other chronic pain), cardiovascular disease (including stroke, heart attack, hypertension), serious long term disease (cancer, diabetes, other serious long term disease), respiratory problems (chronic obstructive bronchitis, emphysema), digestive ulcers, and accident requiring medical attention. Neurological problems were not examined in the present study. Persons were categorized into two groups reflecting the presence or absence of chronic back, lower back or neck pain regardless of other types of comorbid medical conditions. In addition, to control for comorbid conditions, two variables were created: one reflecting the presence or absence of other forms of chronic pain (arthritis/rheumatism, frequent or severe headache and any other type of chronic pain), and one reflecting the presence or absence of any other medical condition (cardiovascular disease (including stroke, heart attack, hypertension), serious long term disease (cancer, diabetes, other serious long term disease), respiratory problems (chronic obstructive bronchitis, emphysema), digestive ulcers, and accident requiring medical attention.

### Quality of life

Quality of life was assessed using the SF-36 Health Survey [22, 23]. This instrument was designed to be applied to any health condition and to assess a person’s functional ability, wellbeing and quality of life. This instrument has been widely used and has been validated in French [24]. The SF-36 has good construct validity, high internal consistency (with Cronbach alphas ranging from .85 to .94 for individual subscales) and high test-retest reliability [24]. It holds eight subscales (vitality, physical functioning, bodily pain, general health perceptions, physical role functioning, emotional role functioning, social role functioning, and mental health) categorized into two scores: the Physical Component Score (PCS) that includes physical functioning, role limitations due to physical health problems, bodily pain and general health, and the Mental Component Score (MCS) that includes vitality, role limitations due to emotional problems, social functioning and mental health. The scores are then transformed into a scale ranging from 0 (reflecting poor quality of life) to 100 (reflecting excellent quality of life).

### Data analysis

First, we present the weighted percentages of chronic back pain by sociodemographic characteristic and test between-group differences (presence or absence of chronic back pain) using chi-square tests. Second, we present the prevalence of other medical conditions among those with and those without chronic back pain. A series of logistic regressions were performed to determine the adjusted odds ratios associated with each medical condition as a function of chronic back pain controlling for sex, age, occupation, education, relationship status, and population density. Third, we present the factors associated with chronic back pain. Multivariable logistic regression analyses were performed to determine the adjusted odds ratios (AORs) and the 95%confidence intervals (CI) for chronic back pain using all variables presented in the table. Fourth, t tests were used to compare the means of quality of life scores between those with and those without chronic back pain. Lastly, multivariable linear regressions beta values were used to determine the association between chronic back pain, sociodemographic characteristics and comorbid conditions and mean quality of life scores. All significance tests were evaluated at the .05 level. All analyses were performed using Stata SE 13.1.

## Results

### Distribution of chronic back pain by sociodemographic characteristic

Table 1 presents the distribution of chronic back pain by sociodemographic characteristic. Overall, the prevalence of chronic back pain is 38.3%.

**Table 1:** Distribution of chronic back pain by sociodemographic characteristic

Sociodemographic variables	No chronic back pain	Chronic back pain	*p*-value
	%	%	
Prevalence	61.7	38.3	
Gender			<.0001
Men	65.7	34.3	
Women	58.7	41.3	
Age-group			<.0001
18–29	73.6	26.4	
30–39	65.7	34.3	
40–49	60.4	39.6	
50–59	55.2	44.8	
60–69	55.9	44.1	
70–79	52.9	47.1	
80–98	54.5	45.5	
Mean age	44.4	49.5	<.0001
Education			<.0001
No degree	55.6	44.4	
Middle school	57.1	42.9	
High school	64.6	35.4	
Some college	66.5	33.5	
College degree	71.6	28.4	
Employment status			<.0001
Employed	63.8	36.2	
Seeking employment	60.7	39.3	
Retired	55.0	45.0	
Student	78.1	21.9	
Homemaker	56.8	43.2	
Other inactive	52.3	47.7	
Occupation			<.0001
Farmer	53.6	46.4	
Independent	56.9	43.1	
Executive	70.4	29.6	
Intermediary profession	62.1	37.9	
Employee	58.6	41.4	
Blue collar worker	58.5	41.5	
Inactive	64.5	35.5	
Relationship status			.001
Single	63.1	36.9	
In a relationship	60.8	39.2	
Population density			<.0001
Rural area	58.4	41.6	
< 20,000	60.1	39.9	
20,000 to 100,000	61.1	38.9	
> 100,000	64.1	35.9	

Note. Weighted row percentages are presented (*n* = 17,224). Chi square tests were performed to identify significant between-group differences. A t test was performed to compare mean age between the two groups

Chronic back pain is found to be more prevalent among women than men (41.3% vs. 34.3%). It also increases significantly with age from 26.4% in the 18 to 29 age group to 47.1% in the 70–79 age group. Education level was found to be inversely proportional to the frequency of back pain. The prevalence of chronic back pain was found to be the highest among farmers (46.4%) and the lowest among executives (29.6%). Employment status was also associated with the prevalence of chronic back pain with the highest prevalence observed among the other inactive category (47.7%) followed by persons in retirement (45.0%). Chronic back pain was reported by 39.2% of adults in a relationship, and 36.9% of single adults. Those living in rural areas or small towns showed a slightly higher rate of chronic back pain as compared to those living in medium or large cities.

### Past year comorbid medical conditions among adults with or without chronic back pain

The majority of adults with chronic back pain (66.6%) reported suffering from another form of chronic pain, and nearly half (43.6%) reported another medical condition (Table 2). Controlling for sociodemographic characteristics including age, persons with chronic back pain had significantly higher odds of reporting arthritis or rheumatism (AOR = 4.76, 95% CI = 4.43–5.13), followed by digestive ulcers (AOR = 2.44, 95% CI = 2.12–2.81), frequent or severe headaches (AOR = 2.31, 95% CI = 2.13–2.62). Chronic back pain was associated with significantly greater odds of each medical condition examined with the exception of diabetes and cancer/leukemia.

**Table 2:** Past year comorbid medical conditions among adults with or without chronic back pain

	No chronic back pain	Chronic back pain		
	%	%	AOR	95% CI
Past year medical conditions				
Any chronic pain	**33.7**	**66.6**	**3.59**	**3.38–3.81**
Arthritis or rheumatism	**15.6**	**45.8**	**4.76**	**4.43–5.13**
Frequent or severe headaches	**14.3**	**26.7**	**2.31**	**2.13–2.62**
Other chronic pain	**11.3**	**21.0**	**1.92**	**1.78–2.07**
Any other medical condition	**29.6**	**43.6**	**1.59**	**1.50–1.69**
Stroke	**0.5**	**1.2**	**2.22**	**1.62–3.06**
Heart attack	**0.6**	**0.9**	1.24	.90–1.70
Hypertension	**12.5**	**19.0**	**1.29**	**1.19–1.40**
Digestive ulcers	**2.5**	**6.5**	**2.44**	**2.12–2.81**
Respiratory problems	**3.6**	**7.0**	**1.77**	**1.56–2.01**
Diabetes	**3.7**	**4.9**	1.11	.97–1.27
Cancer or leukemia	**1.5**	**1.9**	1.04	.84–1.29
Other serious long term disease	**8.0**	**13.0**	**1.61**	**1.47–1.76**
Neurological problems	**2.1**	**4.6**	**2.13**	**1.82–2.50**
Serious accident	**4.5**	**6.1**	**1.41**	**1.24–1.59**

Note. Percentages are weighted. Bold signifies statistically significant chi square tests at *p* = .05 or greater. Adjusted Odds Ratio: Those with chronic back pain are compared to those without and odds are adjusted for sex, age, occupation, education, relationship status, and population density

### Predictors of chronic back pain

After controlling for covariates presented in the table, women were 1.15 times more likely to suffer from chronic back pain as compared to men (Table 3). The risk of chronic back pain increased with age. Education acted as a protective factor as those who completed some college or earned a college degree experienced lower risk of chronic back pain as compared to those without any degrees. Being an independent worker was associated with increased odds of chronic back pain, though none of the other occupations exerted an effect on chronic back pain. People living in urban areas were at lower risk of suffering from chronic back pain than those living in rural areas. Persons in a relationship also had an increased likelihood of chronic back pain. Finally, the presence of another chronic pain condition or any other medical condition significantly increased the odds of reporting chronic back pain.

**Table 3:** Sociodemographic and clinical predictors of chronic back pain

	Chronic back pain	
	AOR	95% CI
Determinants		
Gender: Women (ref: men)	**1.15**	**1.08–1.23**
Age	**1.00**	**1.00–1.01**
Education (ref: no degree)		
Middle school	.98	.88–1.08
High school	.91	.81–1.02
Some college	**.85**	**.75–.97**
College degree	**.71**	**.63–.82**
Occupation (ref: farmers)		
Independent	**1.29**	**1.00–1.67**
Executive	.97	.76–1.23
Intermediary profession	1.15	.91–1.44
Employee	1.08	.86–1.35
Blue collar worker	1.20	.95–1.50
Inactive	.94	.75–1.19
Relationship status: In a relationship (ref: single)	**1.13**	**1.06–1.20**
Population density (ref: rural area)		
< 20,000	**.91**	**.82–99**
20,000 to 100,000	**.88**	**.81–.97**
> 100,000	**.84**	**.80–.90**
Comorbidity		
Any chronic pain (ref: absence)	**3.46**	**3.26–3.68**
Any other medical condition (ref:absence)	**1.32**	**1.24–1.41**

Note. AOR: Adjusted odds ratios obtained in a multivariable logistic regression controlling for all variables presented in the table (*n* = 17,050). Bold signifies statistically significant odds at *p* = .05 or greater

### Quality of life by chronic pain status

Significant associations were found among all physical and mental health indicators with the presence of chronic back pain (Table 4). Persons with chronic back pain scored substantially lower on all SF-36 subscales including both composite physical (PCS) and mental scores (MCS) reflecting a worse quality of life as compared to persons with no chronic back pain. When controlling for sociodemographic covariates and for comorbid conditions, chronic back pain, gender, age, relationship status, education, other types of chronic pain and other medical conditions predicted both lower PCS and MCS scores (Table 5). Occupation appeared to have a stronger association with mental health as compared to physical health.

**Table 4:** Quality of life by chronic pain status

	No chronic back pain	Chronic back pain	*p*-value
Mean scores for SF-36 subscales and composite scales			
Physical functioning	91.48	81.34	<.0001
Role limitation caused by physical health	89.85	75.25	<.0001
Body pain	79.99	55.44	<.0001
General health	76.53	66.76	<.0001
Vitality (energy/fatigue)	64.21	56.81	<.0001
Social functioning	89.57	81.49	<.0001
Role limitation caused by emotional problems	94.67	91.41	<.0001
Emotional well-being	73.24	67.49	<.0001
Physical component score (PCS)	49.43	44.64	<.0001
Mental component score (MCS)	55.97	53.74	<.0001

Note. Mean scores for SF-36 subscales and composite scales. Scores represent the level of functioning and range from 0 (poor) to 100 (excellent). The *p* value indicates the level of significance in the t test used to compare the two groups

**Table 5:** Association of chronic back pain with the Physical Composite Score and Mental Composite Score

	Physical Composite Score			Mental Composite Score		
	Coef	95% CI		Coef.	95% CI	
Chronic back pain status (ref: No pain)	**−2.96**	**−3.13**	**−2.80**	**−1.25**	**−1.45**	**−1.05**
Gender: female (ref: male)	**−.54**	**−.71**	**−.37**	**−1.23**	**− 1.42**	**− 1.03**
Age	**−.08**	**−.08**	**−.07**	**−.02**	**−.03**	**−.01**
Education (ref: no degree)						
Middle school	**1.00**	**.73**	**1.27**	**1.03**	**.70**	**1.36**
High school	**1.42**	**1.10**	**1.74**	**1.16**	**.78**	**1.54**
Some college	**1.50**	**1.17**	**1.84**	**1.28**	**.86**	**1.69**
College degree	**1.50**	**1.15**	**1.84**	**.97**	**.54**	**1.39**
Occupation (ref: farmers)						
Independent	.56	−.13	1.24	**1.27**	**.43**	**2.11**
Executive	**.78**	**.15**	**1.41**	**.83**	**.04**	**1.62**
Intermediary profession	.59	−.02	1.19	**.94**	**.18**	**1.70**
Employee	.19	−.41	.79	**.92**	**.16**	**1.68**
Blue collar worker	−.09	−.69	.52	**.83**	**.07**	**1.59**
Inactive	−.59	−1.21	.04	−.41	−1.21	.39
Relationship status: In a relationship (ref: single)	**.16**	**.00**	**.31**	**1.36**	**1.16**	**1.55**
Population density (ref: rural area)						
< 20,000	.12	−.13	.37	−.14	−.44	.15
20,000 to 100,000	.19	−.06	.44	**−.43**	**−.74**	**−.13**
> 100,000	−.02	−.21	.17	**−.45**	**−.68**	**−.22**
Comorbidity						
Any chronic pain (ref: absence)	**−3.07**	**−3.23**	**−2.90**	**−1.59**	**−1.79**	**−1.40**
Any other medical condition (ref:absence)	**− 2.45**	**−2.62**	**− 2.27**	**−2.52**	**− 2.74**	**−2.30**
R^2^	0.4302			0.1548		

Note. Multivariable linear regression predicting physical and mental composite scores controlling for all socio-demographic variables presented in the table. Bold signifies statistically significant coefficients at *p* = .05 or greater

## Discussion

Chronic back pain is regarded as a major public health issue due to its high prevalence and to its associated economic and social consequences. The present study sought to identify the determinants of chronic back pain and to examine the association of chronic back pain with quality of life in a large population-based survey of French residents ages 18 to 98. The results indicated a high prevalence of chronic back pain and significant burden in terms of quality of life. Importantly, when controlling for other types of chronic pain and for other serious medical conditions, chronic back pain imposed a significantly higher burden for individuals.

In the present study, the overall prevalence of chronic back pain was quite high, reported by 38.3% of the population. In the French portion of the World Mental Health Survey, the prevalence of chronic back pain was found to be 21.3% [3]. An even lower prevalence was reported for France in a European survey [6] and the National Health Survey 2002–2003 [17]. These differences could be explained in part by the question used to evaluate back pain. For example, the European study focused on respondents who had reported pain for more than 6 months, had experienced pain in the previous month and several times during the previous week whereas our study focused on back pain during the past year or currently. Nonetheless, the fact that four out of ten respondents indicated suffering from chronic back pain highlights the importance of this condition in the general population.

Women were at greater risk for chronic pain as compared to men, consistent with evidence that women of all ages experience chronic pain more often than men [3, 25]. The observed gender differences have been linked to the combination of biological, psychological and social factors [26–28]. The findings further confirm that the risk of chronic pain increases with age [28–30]. In the present study, chronic back pain was found to be inversely associated with education confirming the finding of the French National Health survey [31]. Occupational history plays a role in chronic back pain as well. Manual workers and unemployed persons are more likely to experience chronic pain as compared to white collar workers [32]. In the present study, approximately one half of the farmers reported chronic back pain. Farmers were at higher risk of experiencing pain than other groups such as independent professionals, executives, manual workers and inactive persons. However, when controlling for covariates, occupation was no longer a significant predictor of chronic pain with the exception of independent workers. In our study, the level of education seemed to be more pertinent than the occupation status as a risk factor. The occupation categories used in this study relied on national census categories that seem to be insufficiently meaningful in terms of their ability to discern physical labor from non-physical labor. In the U.S., a survey among farmers identified back pain as an occupational health hazard [33]. In another survey among Kansas farmers, the lower back was the anatomical area with the highest prevalence of self-reported work-related pain (37.5%) [34]. Middle-aged farmers were also found to be at higher risk of chronic back pain [35]. It would be useful for future studies to differentiate occupational status according to the physical working constraints and psychological stress constraints [31, 36, 37].

Importantly, chronic back pain was associated with both physical and mental wellbeing even when controlling for comorbid medical conditions and other forms of chronic pain. The findings further showed that back pain was associated with poorer quality of life; it also remained a factor associated with physical and mental health, respectively after controlling sociodemographic factors and comorbid conditions. These findings are consistent with the results of the National Survey in Spain in 2001 using the Health Assessment Questionnaire (HAQ) and SF-12 instruments, lower back pain was found to significantly deteriorate quality of life as well as functioning [38]. In fact, when compared with bipolar disorder, chronic back pain showed similar impairment in mental health and higher physical impairment [39]. Impairment related to chronic pain is often linked to the disruption of daily activities, disability, unemployment, psychological impact and drug abuse [32]. In a case-control analysis from insurance claims data in the U.S., patients with chronic lower back pain had a greater comorbidity burden than the control group which included significantly higher frequency of musculoskeletal pain, neuropathic pain, depression (13.0% vs. 6.1%), anxiety (8.0% vs. 3.4%), and sleep disorders (10.0% vs. 3.4%) [10]. The latter reports are in line with the present findings of significant comorbid medical conditions among those with chronic back pain.

Effective management of chronic back pain remains inadequate. Studies in Western Europe report under-treatment of pain [40]. Even after implementing various changes in legislation and work environment in Finland, the prevalence of chronic back pain has remained the same over the past 15 years [41]. A systematic review of primary care patients in the U.S. revealed that approximately 65% of patients with non-specific lower back pain still experienced pain a year after its onset; a proportion of 41% in Australia and 69% in Europe [42]. In a study of the clinical course of chronic lower back pain and related disability in the Netherlands, approximately 75% of patients whose pain had resolved before the end of their12 month follow-up reported one or more relapses within the following year [43]. Taken together, these findings underscore the need to pursue international data collection in order to evaluate, compare and improve the efficiency of pain management in a global public health perspective [44].

Several limitations should be acknowledged. First, as the data were collected in 2005, it is possible that more recent efforts in diagnosing and treating back pain and other diseases may have affected the prevalence and health-related consequences of chronic back pain. Though the demography has also changed, we found that the proportion of men vs. women is still 48:52 according to 2016 French census (INSEE). The second limitation resides in our decision to categorize neck pain, back pain and chronic back pain as one group when the complexities of these conditions might differ from one another and be associated with different outcomes. Third, the conditions were self-reported without any professional or medical assessment potentially causing biased reporting. Fourth, the assessment of pain did not include information on the duration or the intensity of pain. In addition and by design, the survey did not include institutionalized adults and adults in long-term hospitalization. Targets who were hospitalized at the time they were initially contacted could be contacted only within three-months. Therefore persons who were not discharged within that time frame were not included. The exclusion of these persons is likely to have led to underestimate the prevalence of chronic back pain. Lastly, we do not have health information on non-respondents nor do we know their reason for refusing to participate.

## Conclusions

To our knowledge this is the largest study of chronic back pain and health related quality of life in a population of French residents. According to our findings, chronic back pain is highly prevalent with important variations in its sociodemographic distribution. Due to the important burden associated with chronic pain, it is important to raise awareness among clinicians and health policy makers on the necessity of early diagnosis, proper management and rehabilitation policies in order to minimize the burden associated with chronic pain. In addition, prevention including information and education as well as physical exercise may further contribute to reducing the population burden of chronic back pain [45].

## Abbreviations

CI: Confidence interval
CIDI: Composite International Diagnostic Interview
HAQ: Health Assessment Questionnaire
LBP: Lower back pain
MCS: Mental Component Score
OR: Odds ratio
PCS: Physical Component Score
SF-36: SF-36 Health Survey
WMH: World Mental Health
